# Supplementary material for: Assessment of the Impact of Increased Physical Activity on Body Mass and Adipose Tissue Reduction in Overweight and Obese Children
Source: Children (Basel). 2023 Apr 23;10(5):764. doi: 10.3390/children10050764 (PMC10217524; doi:10.3390/children10050764)
Supplement: Supplementary file 1 [file children-10-00764-s001.zip › children-2306093-supplementary.pdf]

children

Supplementary File S1—Questionnaire

Dear Sir/Madam

I kindly request your answers to this survey questions. The aim of this questionnaire is to determine types of physical activity the families of examined children undertake. The results will be used for purposes of the doctoral dissertation. All questionnaire results are anonymous. I would really appreciate accurate and honest answers as only this type of reply will render survey conclusions reliable.

Primary School……….. Grade …………

Child's first name and surname

Father's age
- 28–40
- 41–50
- 51–60

Mother's age
- 28–40
- 41–50
- 51–60

Father's body mass…………..….body height…………………..

Mother's body mass…………......body height…………………..

Father's education
- Elementary level
- Basic vocational level
- General secondary level
- Higher level

Mother's education
- Elementary level
- Basic vocational level
- General secondary level
- Higher level

Your child spends its free time on
- watching TV/ in front of the computer, reading
- physical activity

Your family participates in physical activity
- sporadically
- once a week
- twice a week
- every day

How often does your child take part in an organised form of physical activity?
- never
- once or twice a week
- three to five times a week
- more often

Consuming fast food
- never
- once a month
- once a week
- more often

Marta Nowaczyk
Poznan University of Physical Education
